# New model for long-term investigations of cutaneous microcirculatory and inflammatory changes following irradiation

**DOI:** 10.1093/jrr/rru124

**Published:** 2015-02-16

**Authors:** Ole Goertz, Christoph Poettgen, Azarm Akbari, Jonas Kolbenschlag, Stefan Langer, Marcus Lehnhardt, Martin Stuschke, Leon von der Lohe

**Affiliations:** 1 Department of Plastic and Hand Surgery, Burn Center, BG-University Hospital Bergmannsheil, Ruhr University Bochum, Buerkle-de-la-Camp-Platz 1, 44789 Bochum, Germany; 2 Department of Radiotherapy, University Hospital Essen, University Duisburg-Essen, Germany

**Keywords:** ionizing radiation, intravital fluorescent microscopy, microcirculation, leukocyte–endothelium interaction, radiotherapy, irradiation

## Abstract

Radiotherapy is used for curative and palliative treatment. However, its negative effect on normal tissue is a limiting factor for the deliverable dose. Microcirculatory breakdown and prolonged inflammation in particular are major features of late side effects. The purpose of this study was to develop a reliable animal model that will allow a long-term *in vivo* analysis of microcirculation and inflammation following irradiation. A single dose of 90 Gy was delivered to the ears of hairless mice (*n* = 15). Intravital fluorescent microscopy was used to assess microcirculatory parameters and leukocyte behaviour. Values for the identical (control) areas were obtained before as well as during the following days, weeks and months following irradiation. The arteriolar and venular diameter increased up to Day 14, decreased during the following months, and increased again after one year. The red blood cell velocity increased up to 145% on Day 3, decreased on Day 7 to 115%, and stayed above baseline value the whole year. The integrity loss of the endothelium increased up to Day 7 and continued up to Day 75 after radiation. After one year, the oedema was at the baseline level. Leukocytes showed their maximal activity at one year after trauma. An increase was measured up to Day 25; the lowest values were measured at Day 40 post-irradiation, followed by a repeated increase. The present model allows a certain visualization of microcirculatory disturbances and inflammation over a period of months. This permits the possibility of long-term investigations of the underlying pathophysiology following irradiation, including possible drug interactions.

## INTRODUCTION

Radiotherapy has curative or palliative potential in about half of all incident solid tumors. Despite the known benefits of irradiation, it does not attack cancer cells only, which limits the deliverable dose [1, 2]. While early side effects in normal tissue arise over a period of weeks to months—including erythema, oedema, inflammation, and moist desquamation—late complications arise after months, years or decades, and include atrophy, pigmentation changes, teleangiectasia, induration, fibrosis, and ultimately ulceration and necrosis [3, 4].

The main reason for the progression of tissue damage is based on prolonged inflammatory processes with activated leukocytes and the continuing impairment of microcirculation [5, 6].

Our aim was to visualize the microcirculatory changes following irradiation over a period of months in order to broaden the morphological knowledge about radiation-induced alterations by dynamic investigations. Therefore, we sought to develop an animal model that allows reproducible long-term *in vivo* analysis of the microcirculation and leukocyte–endothelium interaction following ionizing radiation.

## MATERIALS AND METHODS

### Animal preparation

The caudal half of the right ear of 15 male hairless mice was used (SKH-1/h, *n* = 15, bodyweight 19–22 g; Charles River, Sulzfeld, Germany). All mice underwent single housing in standard polycarbonate cages (21°C, 12 h dark/light cycle). The animals had access to standard laboratory chow and tap water *ad libitum*. Each procedure was approved by the regional authorities according to German animal care regulations, which comply with the international guidelines of animal care and use in scientific experiments (AZ: 50.8735.1, Nr.: 112/5). After finishing the experiments, the animals were put down under general anaesthesia by an overdose of pentobarbital.

Mice were anaesthetized by spontaneous inhalation of isoflurane-N_2_O (F_i_O_2_ 0.35, 0.015L/L isoflurane, Forenetized by spontanWiesbaden, Germany) and placed on a heated acryl-glass observation platform to maintain a body temperature of 37 in scientific experiments (AZ: 50.8735.1, Nr.: 112/5). After finNorderstedt, Germany) were pulled through the ear for extension. In addition, physiological saline between ear and platform was used to flatten the ear by adhesive forces.

Fluorochromes were administered via the tail-veins (tube 29G, Braun, Melsungen, Germany): 25 µl FITC-labelled dextran (1.0%, MW 150 KDa) served as a plasma marker, and 25 µl rhodamine 6G (0.5%) was used for *in vivo* labelling of leukocytes (both: Sigma Chemicals Co., St Louis, MO, USA).

### Irradiation

We used an Orthovolt X-ray tube for radiation delivery. Radiation was delivered on an area of 1.3 mm^2^ on the dorsal side of the ear using a Philips X-ray generator (RT 100^(R)^, 45 kV, 10 mA; applicator: 1 cm diameter; source–skin distance: 10 cm; filter: 0.55 mm Al; dose rate: 9.4 Gy/min). Mice were positioned in a semi-circular perspex shell, allowing for application of inhaled narcotics and continuous monitoring of animals. The ear was placed laterally on a flat plane. Fixation was done with microsurgical loops and rubber strips. A custom-built lead shield with a circular aperture was placed over the appropriate tissue area. Using a Philips X-ray generator, we administered a total dose of 90 Gy in one fraction.

### Recordings

Before irradiation, the prospective area was designated and the microcirculatory parameters of the regions of interest (ROIs) were assessed to achieve an intra-individual comparison of the data. For the intravital fluorescence microscopy (Axiotech vario, Carl Zeiss, Oberkochen, Germany), we used a 4-fold and a 20-fold water immersion objective (Achroplan x4/x20, Zeiss). We determined six regions of interest (ROI, minimum two arterioles and two venules in each region) in and around the irradiation area. Our baseline values were derived from the recordings directly before radiation injury; the data being given in percentages permitted focus on proportionality changes. The *x*-*y* coordinates of all the measuring regions were saved, and digital pictures were taken for image matching of anatomical structures to ensure relocation of the areas throughout the study.

The images were recorded using a charge-coupled video camera (AVT-BC 71, AVT-Horn, Aalen, Germany) and stored digitally. Microscopic observations were performed prior and on Days 1, 3, 7, 14, 25, 40, 75 and 365 after irradiation. The period of narcosis lasted ∼45 minutes on each observation day.

### Measurements

The microcirculatory parameters were quantified with an off-line computer-assisted image analysis system (CapImageoughout the study.iaHeidelberg, Germany). Diameters of arterioles and venules were measured in micrometers. The venular red blood cell velocity (Venular RBCV, mm/s) was calculated using the analysis system and was only measured in venules. Venular blood flow (fl/s) was calculated using a standard formula in microcirculation [7]. Because of the calculated parameter, we did not perform any statistical tests to assess possible significant differences. The ratio of extravascular versus intravascular intensity showed oedema formation, with a consecutive leakage of the plasma marker FITC-dextran from the vessels into the surrounding tissue. The leukocyte–endothelial interaction was analyzed using two different parameters: the number of intermittent adherent leukocytes (such as rolling leukocytes) passing a reference point within the venule per minute (n/min), and the number of permanent adherent leukocytes (such as leukocytes that stick to the venular endothelium) in a defined length per minute (n/100 µm/min).

The ears were excised after finishing the experiments, affixed to a piece of cork, placed in 5% neutralized formalin, paraffin-embedded and frozen at −20°C for cross sectioning. The sections were de-paraffinized and stained with hematoxylin and eosin.

### Statistics

The commercially available computer program SPSS version 21 (SPSS GmbH, Munich, Germany) was used for statistical analysis of the data. The statistical evaluation was performed in a blinded manner. During the evaluation of the microcirculatory changes with the analysing system the investigator did not know about the day of recording. Out of a single set of data, the mean value, the standard error, and the standard error of the mean of each mouse were calculated. To compare the different values with one another, a variance analysis for repeated measurements was used. The mean value of the significant data was compared with the *t*-test for paired samples. A *P-value* < 0.05 was considered statistically significant.

## RESULTS

The number of animals was reduced from 15 to 10 after Day 14 because of harvesting tissue samples for hematoxylin and eosin staining. After harvesting, the small area did not allow for further intravital microscopic evaluations.

**Fig. 1.**
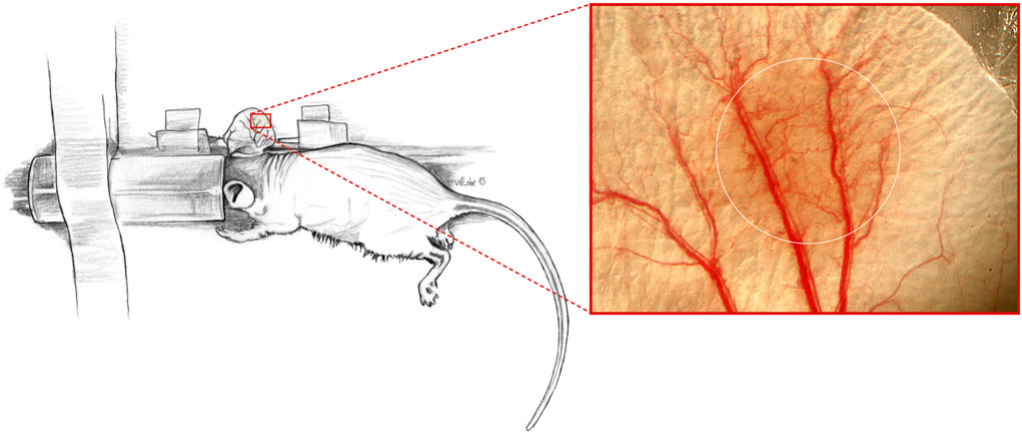
Macroscopic picture of the ear 25 days after radiation injury using conventional digital technique. The area of irradiation reddened, the microvessels seem to be dilated. The white ring marks the area of irradiation.

The macroscopic evaluation of the radiated area showed paleness immediately after injury; in the periphery of the area, the skin reddened after a few minutes. The intensity of the redness increased during the following days and weeks and regressed slowly (Fig. 1).

**Fig. 2.**
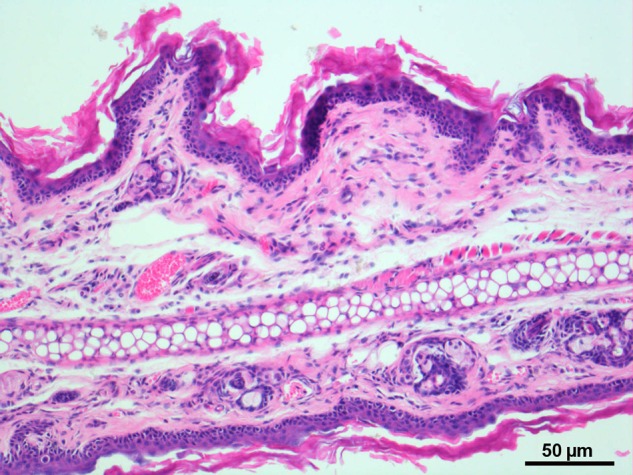
The histological section in HE-staining showed oedema and in parts necrosis of the epidermis and partial dermis 25 days post irradiation. The number of immigrated leukocytes in the histological sections was most pronounced 25 days post trauma.

Microscopic, histological observation

revealed oedema and necrosis of parts of the epidermis and dermis 25 days post irradiation. The number of immigrated leukocytes in the histological sections was pronounced the most at 25 days post trauma (Fig. 2).

**Fig. 3.**
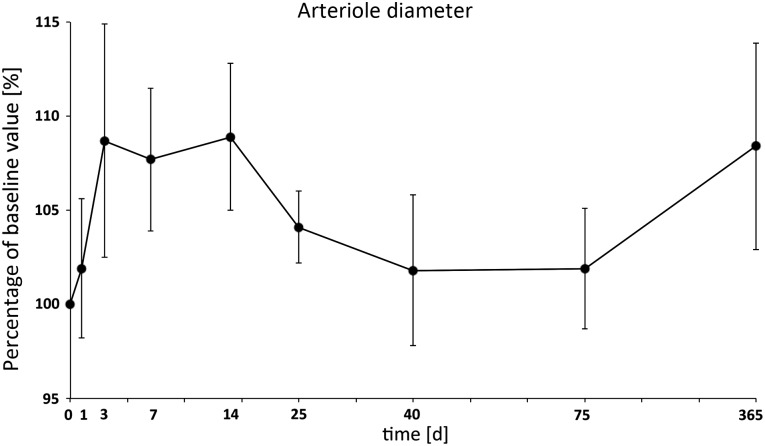
The arterial diameter increased by a factor of 1.1 directly post radiation injury and showed permanently increased dilatation up to Day 14, when its maximum was reached. It was reduced to nearly normal values the following weeks, with another increase one year post radiation injury.

The arterial diameter rose to a factor of ∼1.1 directly post irradiation, and its dilatation rate permanently increased up to Day 14, when its maximum was reached at 109% of baseline value. During the following weeks, the dilatation rate was nearly within normal ranges. Another increase was detectable 1 year post irradiation (Fig. 3, Table 1).

**Table 1.** Microcirculatory parameters of the treatment group (*n* = 15) from Day 25 (*n* = 10)

	Day 1	Day 3	Day 7	Day 14	Day 25	Day 40	Day 75	Day 365
Arteriole diameter (%)	101.9 ± 3.7	108.7 ± 6.2	107.7 ± 3.8	108.9 ± 3.9	104.1 ± 1.9	101.8 ± 4.0	101.9 ± 3.2	108.4 ± 5.5
Venular diameter (%)	104.3 ± 2.5	105.0 ± 3.8	106.3 ± 4.0	111.3 ± 2.3	107.0 ± 3.0	110.3 ± 4.9	105.2 ± 3.1	111.2 ± 5.5
Venular RBCV (%)	120.5 ± 11.0	145.0 ± 15.3	114.5 ± 8.2	124.4 ± 11.0	127.9 ± 15.0	128.3 ± 14.9	124.5 ± 15.0	129.3 ± 19.8
Venular blood flow (%)	132.2 ± 13.6	171.2 ± 27.8	133.6 ± 16.3	157.7 ± 16.3	152.4 ± 27.0	170.0 ± 35.1	139.8 ± 19.3	178.9 ± 48.2
Oedema (%)	100.3 ± 3.7	109.8 ± 3.8	125.7 ± 7.5	120.6 ± 5.0*	124.2 ± 11.3	121.1 ± 12.0	114.8 ± 6.0	93.1 ± 3.1
Roller (%)	111.7 ± 9.7	130.3 ± 11.7	120.9 ± 13.0	86.8 ± 9.1	110.9 ± 14.7	79.2 ± 14.5	110.1 ± 14.7	210.6 ± 24.0*
Sticker (%)	143.0 ± 39.4	133.0 ± 15.0	167.6 ± 35.2	185.5 ± 47.1	190.6 ± 36.1*	109.5 ± 25.8	169.9 ± 40.7	246.6 ± 69.0

Data are expressed as a percentage compared with the baseline value ± SEM. RBCV = red blood cell velocity, asterisk indicates *P* < 0.05 vs baseline value. The number of animals was reduced from 15 to 10 after Day 14 because of harvesting tissue samples.

The venular diameter also increased directly post trauma, but stayed above the baseline value throughout the entire observation period. The maximal dilatation was measured on Day 14, with 111% of the baseline value (Table 1).

The venular RBCV showed its maximum on Day 3 at an increase reaching 145% of the baseline. Lowest values were measured on Day 14 (115%), and this was followed by another increase. After one year, values of ∼130% were measured (Table 1).

The venular blood flow showed a quite similar tendency in values. After a peak on Day 3 at 170%, it decreased to 130% on Day 7, then started to increase again. Highest values were measured one year after irradiation, with nearly 180% of baseline (Table 1).

**Fig. 4.**
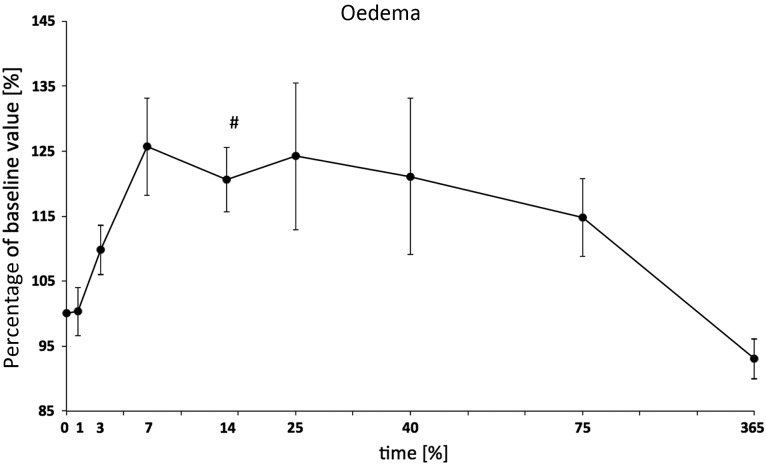
Oedema formation due to extravasation of FITC-dextran in the surrounding tissue. The increase started on Day 3 and reached its maximum on Day 7 post irradiation (with 125% of the baseline value). The increase was followed by a continuous decrease; the lowest values were measured one year after irradiation. From Day 3 to Day 75 it stayed high above the baseline.

The leakage of the plasma marker FITC-dextran into the extravasal tissue was used as a parameter for the oedema formation. The increase started at a later point in time (Day 3) and reached its maximum on Day 7 post trauma at 125%. The increase was followed by a continuing decrease; the lowest values were measured one year after trauma. From Day 3 to Day 75 it stayed high above the baseline (Fig. 4, Table 1).

The number of rolling leukocytes on the endothelium increased following the irradiation up to Day 3. The maximum was measured one year after irradiation, at 210% of the baseline number (Table 1).

**Fig. 5.**
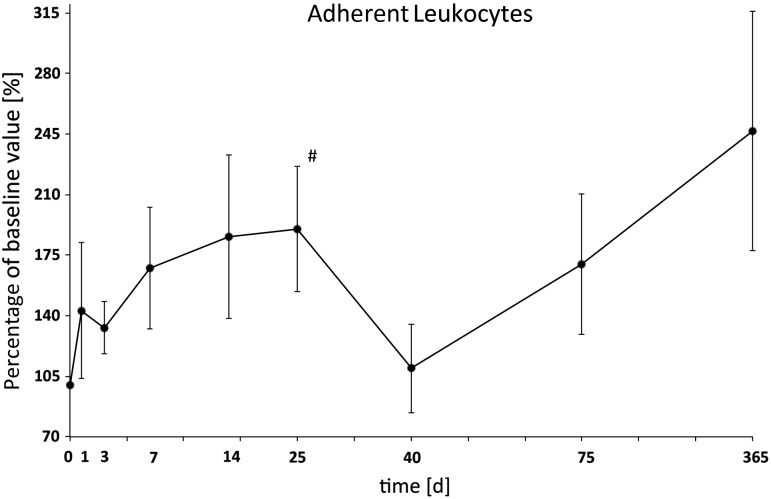
Adherent or sticking leukocytes on the venular endothelium. The number increased up to Day 25, followed by a decrease on Day 40. The number increased again to 170% on Day 75 and was nearly 250% one year after irradiation.

The number of sticking or adherent leukocytes increased up to Day 25, followed by a decrease till Day 40. The number increased again to 170% on Day 75, and to nearly 250% one year after irradiation (Fig. 5, Table 1).

## DISCUSSION

Radiotherapy is a powerful tool with which to treat a range of types of cancer [2, 8]. The higher the dose, the better the intended destructive effect on the tumour. However, normal tissue tolerance may limit unrestricted escalation of the dose [2, 9]. Besides early side effects, late complications such as tissue ulceration and fibrosis (with malfunctions of the affected organs) are limiting factors [2, 3, 9]. The main reason for the progress of tissue damage over an extended period lies in the prolonged inflammation–leukocyte activity together with the continuing impairment of the microcirculation, mainly due to DNA lesions [2, 5, 6]. To allow long-term *in vivo* investigations of the inflammatory process and the microcirculation, we have developed the current model. Since no invasive preparations that would easily harm the model are performed, this model allows repetitive *in vivo* investigations of the same vessels and quantitative leukocyte investigations over the months following irradiation.

Previously developed radiation models allowing *in vivo* investigations used the cremaster muscle, the mesentery, the pia mater and the retina of the rat, and the ileum of mice [5, 10–14]. Other models deal with immunohistochemistry and morphomectric analyses in rats [15] or pigs [16].

In our model, the focus is on the dermal and subcutaneous tissue. This facilitates the comparison of the effects of different physical injuries on superficial tissues [17]. Easily accessible *in vivo* models are of great importance, especially for study of the regenerative processes of vascular architecture, not only in injury investigations, but also in tumour biology studies [18–20].

We did not determine the functional vessel density. It turned out that it was mainly the quality of the recordings that was responsible for the results and not the actual vessels. When the oedema formation is pronounced, as it was in the present investigations, the contrast between intra- and extravasal tissue is abolished. Very small vessels in particular cannot be evaluated reliably.

A study using the retina of rats following 10 Gy irradiation revealed a continuous decrease of the vessel diameter up to Day 60, when the observations ended [5]. Our results are in contrast to these findings, even when much higher doses were used. Baseline values of the arteriolar and venular diameter were exceeded at every point in time in both the venules and the arterioles. We interpret these findings as partly due to the inflammation process, which could also be observed in the loss of integrity of the endothelium, as well as in the increased leukocyte–endothelium interaction.

The arteriolar RBCV was not measured because the accuracy of measurement is not acceptable in velocities over 0.7 mm/s, as previously demonstrated [20]. This speed was regularly exceeded.

The venular RBCV showed an increase on Day 1 post irradiation, followed by a reduction of the velocity, but the level was always above baseline during the whole observation period. In combination with the increased diameter, this indicates a higher venular blood flow as part of the higher metabolic activity. Siemionow *et al.* was able to demonstrate, in the cremaster muscle of rats, that low-dose radiation (8 Gy) causes an increase in vessel diameter and RBCV in first- and second-order arterioles, with highest rates at 72 h and one week, while third order arterioles were constricted. However, none of these findings were statistically significant [11]. We observed similar findings: 72 h after irradiation there was a peak, and further peaks were reached at Day 40 and Day 365. These findings allow us to hypothesize that post-radiation inflammation is divided into two parts: a fast reaction (after 72 h) and long-term injury, probably as part of the endothelial damage [2].

The integrity loss of the endothelium post radiation, with subsequent oedema formation, had a delayed beginning on Day 3 and lasted in our study until Day 75.

The FITC-dextran in the extravascular tissue is not only a result of new oedema formation, but also of residues from the accumulated FITC-dextran due to the repeated injections, especially during the first week (when many measurements were performed) [21]. However, the later results could be fully attributed to the integrity loss, because the last injection was about one month earlier. The oedema formation and the decreasing intracellular and intravascular fluid results in compromise of cellular respiration and decreased tissue perfusion, which enhances tissue damage [22, 23]. Changes in permeability after radiation-induced damage were also described for the pia mater and cremaster muscle [12].

The analysis of leukocyte–endothelium interactions was performed in venules with a diameter of between 15 and 30 µm. An increase in leukocytes rolling and sticking to the inner vessel wall was noted on Day 1. While the quantitative upregulation of the leukocytes continued only up to Day 25, their activation rate was elevated during the complete period of observation. The highest number of rolling as well as sticking leukocytes was measured after one year, indicating a long-lasting inflammatory process and a further increase till most of the wounds would already be healed. The rolling process reduces the velocity and simultaneously allows time for leukocytes to detect chemotactic signals on the endothelial surface. The rolling and sticking process represents the first two steps for immigration of the leukocytes into the surrounding tissue [21]. The increasing number of leukocytes plays an important role in the damage progression of irradiated tissue [5]. After radiation, activated neutrophils also produce oxygen free radicals, which also trigger tissue damage [13]. An *in vitro* study using a parallel-plate chamber and real-time video-microscopy revealed that irradiation (10 Gy) already affects the endothelial cells. Rolling as well as sticking of leukocytes was increased, and pro-inflammatory and thrombogenic responses were enhanced [24]. An *in vivo model* using the retinal microcirculation of rats following a 10 Gy irradiation showed a continuous increase in leukocytes starting on Day 1 after radiation and which continued for the whole observation period up to Day 60 [5].

Siemionow *et al.* demonstrated in rats that low-dose radiation (8 Gy) had no influence on leukocyte–endothelial interaction at all [11]. In the mesentery of rats, an increase in leukocytes and oxygen free radicals could be observed 2 and 6 h after irradiation [13]. Irradiation of the ileum of mice with a dose of 19 Gy caused an increase in rolling leukocytes (with a peak after 2 h) and an increase in adhesive leukocytes (with its maximum 16 h post radiation). Leukocyte rolling and adhesion were back to baseline 48 h after radiation [10].

Molla *et al.* demonstrated that the endothelial P-selectin expression was upregulated following irradiation (4 and 10 Gy, respectively), and that this was paralleled by an increase in the number of rolling leukocytes. In contrast, in P-selectin–deficient mice, an increase in rolling leukocytes did not occur, but the number of sticking leukocytes and the tissue damage was similar to that of wild-type mice [14]. These findings strongly indicate that the first step of leukocyte-migration can be skipped and that the isolated neutralization of P-selectin cannot reduce radiation-induced inflammation [14]. Additionally, the same author demonstrated that the intercellular adhesion molecule 1 (ICAM-1) plays a key role in leukocyte adhesion in the earlier stages (first 24 h) and that vascular cell adhesion molecule 1 (VCAM-1) plays a key role at a later stage (after 14 days) in the inflammatory response following irradiation [25].

## CONCLUSION

In summary, the present irradiation model can be considered as both reliable and reproducible. Studies of microcirculation, inflammation and leukocyte–endothelium interactions, always returning to the same blood vessel, are feasible over observation periods from minutes to years, allowing visualization and investigation of early, intermediate and long-term effects of radiation injury. Moreover, this model could enhance our understanding of the underlying pathophysiological mechanisms that result in histological and molecular changes.

## CONFLICT OF INTEREST

My coworker and I herewith certify that there are no financial or proprietary interests in the subject matter or materials discussed in this manuscript.

## FUNDING

Funding to pay the Open Access publication charges for this article was provided by the “Ruhr-University Bochum” and “Deutsche Forschungsgemeinschaft (DFG)”.
